# Lightweight Visual Odometry for Autonomous Mobile Robots

**DOI:** 10.3390/s18092837

**Published:** 2018-08-28

**Authors:** Mohamed Aladem, Samir A. Rawashdeh

**Affiliations:** College of Engineering and Computer Science, University of Michigan-Dearborn, Dearborn, MI 48128, USA; maladem@umich.edu

**Keywords:** visual odometry, ego-motion estimation, stereo, RGB-D, mobile robots

## Abstract

Vision-based motion estimation is an effective means for mobile robot localization and is often used in conjunction with other sensors for navigation and path planning. This paper presents a low-overhead real-time ego-motion estimation (visual odometry) system based on either a stereo or RGB-D sensor. The algorithm’s accuracy outperforms typical frame-to-frame approaches by maintaining a limited local map, while requiring significantly less memory and computational power in contrast to using global maps common in full visual SLAM methods. The algorithm is evaluated on common publicly available datasets that span different use-cases and performance is compared to other comparable open-source systems in terms of accuracy, frame rate and memory requirements. This paper accompanies the release of the source code as a modular software package for the robotics community compatible with the Robot Operating System (ROS).

## 1. Introduction

Accurate and real-time motion estimation to enable robot perception and control tasks is a fundamental problem in mobile robotics. Despite being well studied, it remains challenging to provide timely and accurate robot motion estimates for applications where the mobile robot is operating in uncontrolled and previously unknown environments. Examples of such applications include micro-aerial vehicles (MAVs) exploring a new environment, advanced driver-assistance systems (ADAS) in modern automobiles and autonomous self-driving vehicles.

A mobile robot’s position estimation can be performed through dead reckoning using a combination of wheel odometers in ground vehicles and inertial measurement units (IMUs). However, continuous integration of measurements from these sensors results in drift in position estimates, rendering them unsuitable for sustained long-term operation. Sonar and ultrasonic sensors can be used for robot localization; however, they are active sensors and can interfere with each other. The global positioning system (GPS) provides absolute position with no error accumulation over time. However, GPS sensors are unavailable in indoor and closed areas and they lose satellite lock in tunnels and urban canyons.

Cameras are attractive sensors for performing the motion estimation task. Cameras are low-cost sensors and can provide a rich stream of information. Furthermore, since cameras are passive sensors, they do not interfere with each other when multiples are deployed. Visual odometry (VO) is the process of estimating the position and orientation of an agent (e.g., a vehicle) using only the input of a single or multiple camera attached to it [1].

In this paper, we present our innovative visual odometry system called lightweight visual tracking (LVT). Unlike typical visual odometry approaches where features are tracked and motion is estimated between consecutive frames only, our system tracks features for as long as possible. This results in a system that is approaching full visual simultaneous localization and mapping (V-SLAM) systems in terms of accuracy while still maintaining low computational overhead. Moreover, the system supports both stereo and RGB-D cameras. For the benefit of the community, the full source code of the system is made publicly available under a permissive license and with support for the Robot Operating System (ROS) at: (https://github.com/SAR-Research-Lab/lvt).

## 2. Related Work

Visual odometry is an active area of research where many different methods have been developed over the years. A detailed review of the field of visual odometry was published by Scaramuzza and Fraunhofer [1]. The problem of estimating vehicle motion from visual input was first approached by Moravec [2] in the early 1980s. Moravec established the first motion-estimation pipeline, whose main functional blocks are still used today. However, the term visual odometry was first coined by Nister et al. in their landmark paper [3]. Their paper was the first to demonstrate a real-time long-run implementation with a robust outlier rejection scheme.

Kitt et al. presented a visual odometry algorithm based directly on the trifocal geometry between image triples [4]. Howard has presented a visual odometry system in which inliers are detected based on geometric constraints rather than on performing the more commonly used outlier rejection schemes [5]. However, those approaches follow the typical visual odometry scheme of matching or tracking features between consecutive frames and using these features for ego-motion estimation. The first to propose using the whole history of tracked features is the work by Badino et al. [6]. They do so by computing integrated features, which are the sample mean of all previous measured positions of each feature. Integrated features are then used in the motion computation between the two consecutive frames. Our approach differs in that we employ a transient local 3D map for tracking. It consists of a sparse set of 3D points (features) that are used for tracking and, therefore, motion estimation. This local map is internal to the system and is not an attempt to build a global map of the environment. As long as a feature is useful and can be utilized for motion estimation, it is kept alive in this local map.

Related to visual odometry is the simultaneous localization and mapping (SLAM) problem, where the goal is to build an accurate map of the environment while simultaneously localizing within this map. Visual SLAM systems employ sophisticated techniques to improve their accuracy, such as detecting loop-closures, where the system detects a previously visited location and uses this information to correct the map. Early work of bringing vision into SLAM was Davison’s MonoSLAM [7], which was a filter-based method utilizing an Extended Kalman Filter (EKF). Filter-based methods were dominant until the development of the Parallel Tracking and Mapping (PTAM) system by Klein and Murray [8]. PTAM separated and parallelized the motion estimation and mapping tasks through a keyframe-based architecture while employing bundle adjustment [9]. An excellent introduction and survey of keyframe-based visual SLAM is by Younes et al. [10]. Unlike typical visual SLAM systems, by employing our local map approach our system is able to achieve high accuracy while maintaining low memory and computation requirements.

Additionally, Visual SLAM and odometry systems can be classified as either direct or feature-based methods. Feature-based methods try to extract distinctive interest points and track them in subsequent frames to estimate camera ego-motion. In contrast, direct methods operate on the whole image directly. That is, the camera is localized by optimizing directly over image pixel intensities. Key representatives of feature-based visual SLAM systems are S-PTAM [11] and ORB-SLAM [12], whereas a representative of the direct visual SLAM systems is LSD-SLAM [13]. Our system is based on point or corner-like features as they enable real-time performance while running completely on a CPU.

With the introduction of commodity depth sensors, RGB-D cameras have become popular in robotics. Huang et al. have introduced a visual odometry system based on RGB-D cameras called Fovis [14]. Their system extracts point features from the image and then each feature’s depth is extracted from the depth image. They follow an inlier detection approach similar to Howard’s [5] and track features between consecutive frames. On the other hand, DVO by Kerl et al. [15] is a dense visual odometry method that aims to exploit both the intensity and depth information of RGB-D cameras. Yet other approaches use depth information alone for ego-motion estimation. One common approach is by 3D point-cloud registration, which is commonly performed using an iterative closest point (ICP) algorithm [16,17]. KinectFusion by Newcombe et al. [18] is one of earliest and most well-known RGB-D SLAM systems. It fuses depth data into a volumetric dense model that is used for tracking camera motion. Our system supports RGB-D data as well, where features are detected from the RGB image and depth information is extracted from the depth image. The rest of the system remains intact and performs the same operations regardless of the used sensor.

## 3. System Description

A high-level overview of the visual odometry algorithm is shown in Algorithm 1. In the rest of the paper, camera pose is understood to encompass both position and orientation, that is, the complete 6 degrees-of-freedom transformation. The world reference coordinate frame is set at the pose of the first frame of the sequence.
**Algorithm 1.** Visual Odometry Algorithm Overview**for** each frame (stereo or RGB-D) Extract features (AGAST, BRIEF) **if** first frame  Initialize local map  Set world reference coordinate frame **else**  Predict new pose  Track local map  Estimate pose using tracked measurements  Update staged map points  Perform new triangulations if necessary  Clear no longer trackable map points **end if** **end for**

The details of different components are illustrated below.

### 3.1. Image Sequence

The first step is to feed the system a frame that is acquired either from a stereo or RGB-D sensor. In the stereo camera case, the retrieved stereo frame consists of the synchronized images from the left and the right cameras. The stereo frame is assumed to be stereo-rectified. Stereo rectification is the process of virtually transforming the stereo frame so that it appears as if the two cameras of the stereo rig have their image planes aligned to be coplanar. Consequently, epipolar lines are now parallel to the stereo baseline between the cameras. This reduces the stereo correspondence problem to a one-dimensional search, as matching features between the two cameras will lie on the same pixel row, assuming a horizontal stereo rig [19]. In the RGB-D sensor case, the RGB image is converted into a grayscale one and then it is fed along with the depth image to the system.

### 3.2. Feature Extraction

As a feature-based method, salient point or corner-like features will be extracted and used for subsequent processing. Corner-like features are fast to compute and many good corner detectors are available. In this work, the adaptive and generic accelerated segment test (AGAST) [20] corner detector is used. AGAST builds on the features from accelerated segment test (FAST) [21] corner detector, which enjoys a high degree of repeatability and computational performance. AGAST improves the accelerated segment test that underlies FAST by making it more generic while increasing its performance. No scale pyramid of any image is built and the corners are extracted from the full-size images.

For each detected feature, a feature descriptor is computed. A feature descriptor acts like a signature for that feature. Matching between features can then be performed by comparing their descriptors. In this work, binary robust independent elementary features (BRIEF) [22] descriptors are used. BRIEF descriptors are binary feature descriptors, that is, descriptors that are in the form of a binary string. This means that matching is fast, based on hamming distance. Hamming distance is defined as the number of positions at which the corresponding bits are different. Hamming distance is simple and fast to compute because it is just an exclusive-OR (XOR) operation and a bit count, that is, sum(xor(descriptor1, descriptor2)). However, BRIEF descriptors lack rotational invariance.

An important problem to consider is the distribution of the detected features across the image. Poor distribution where detected features are concentrated in one region of the image can lead to poor results. We follow a two-step process to overcome this problem. First, the image is divided into cells where features will be detected in each cell separately. Then, a technique known as adaptive non-maximal suppression, as described by Brown et al. [23], is performed in each cell. Adaptive non-maximal suppression aims to limit the maximum number of features extracted while at the same time ensuring good distribution across the image. Features are suppressed based on corner strength and only ones that are local maxima in the neighborhood are retained.

### 3.3. Pose Prediction

Before proceeding to the next step, a prediction of the current camera pose is performed. For ground vehicles, dead reckoning using wheel odometry can be used to perform such prediction since it is usually available. In this work, we will follow a simple motion model where velocity is assumed constant between frames and then computed velocities are averaged over frames. In this simple motion model, a constant frame rate is assumed, that is, time is not considered in the calculations. The motion is calculated as follows: assuming the pose at frame k to be $C_{k}$, which consists of orientation represented as a quaternion, $q_{k}\in SO\left(3\right)$ and position $t_{k}\in \;{\text{ℝ}}^{3}$. $C_{k}$ is the pose to be predicted. The linear velocity at frame k is computed as:

(1) $$v_{k}=\;t_{k-1}-t_{k-2}$$

This linear velocity is then averaged over time:

(2) $$v_{k}=\left(v_{k}+\;v_{k-1}\right)/2$$

A similar approach is followed for the rotational component where quaternion multiplication is performed:

(3) $$w_{k}=\;q_{k-1}q_{k-2}^{-1}$$

and then the rotational velocity is also averaged over time using the spherical linear interpolation (SLERP) operation:

(4) $$w_{k}=slerp\left(w_{k},\;w_{k-1},\;0.5\right)$$

After computing the new velocities, the predicted pose is easily computed as follows:

(5) $$t_{k}=\;t_{k-1}+\;v_{k}$$

(6) $$q_{k}=\;q_{k-1}w_{k}$$

The predicted pose is then used as a guide in the next local map-tracking step.

### 3.4. Tracking Local Map

**Figure 1:**
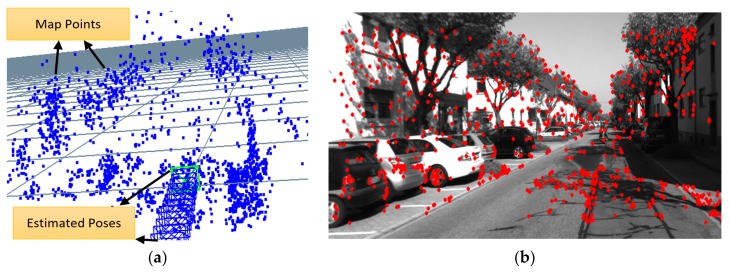
An illustration showing: (**a**) local 3D map and (**b**) detected 2D image features. The goal is to find the correspondence between each 3D map point and detected 2D feature. Sample image from KITTI dataset.

The goal of this step is to correctly associate visible 3D map points with 2D image features. This 3D-2D data association is used by the following pose estimation step. Note that the associated 2D image features here are, in the stereo camera case, the ones extracted from the left camera image only. The 3D map points are projected onto the image plane of the left camera in the stereo camera case and of the RGB camera in the RGB-D camera case using the pinhole camera model. The neighborhood of each projected 3D map point is then examined for the best matching 2D feature detected in the feature extraction step. In our current implementation, the search neighborhood is set to a 25-pixel radius around each projected feature. If no enough matches are found, the search radius is doubled and the tracking step is performed again. This process is illustrated in Figure 1, where the local 3D map is shown to the left and the detected 2D image features in the current frame are shown to the right.

Neighborhood search is accelerated by means of spatial hashing. During the feature extraction step, the image plane is divided into a two-dimensional grid. Each cell of this grid is assigned the list of corners that happen to be within its boundaries. Now, the projected point’s prospective grid cell is computed and thus the list of potential matches is readily available. Once candidate neighborhood features are identified, finding the best match of the projected point proceeds by using the widely accepted ratio test proposed by Lowe [24]. The ratio test works by comparing the distance of the closest neighbor to that of the second closest one. The nearest neighbor here is defined as the one with the minimum hamming distance for the BRIEF descriptor. If the nearest feature is much closer than the second nearest one, then it has a higher probability of being the correct match. The ratio test value is set to 0.80 in our implementation.

### 3.5. Pose Estimation

The found matches are then used for computing the camera pose. Camera pose consists of orientation R∈SO(3) and position $t\in {\text{ℝ}}^{3}$. Finding the camera pose is formalized as an optimization problem to find the optimal R,t that minimizes the reprojection error between the matched 3D points and the image 2D features:

(7) $$\left\{R,t\right\}=\underset{R,t}{\mathrm{argmin}}\underset{i\in S}{\sum }\rho \left(||x^{i}-\pi {\left(RX^{i}+t\right)||}^{2}\right)$$

where $x^{i}\in {\text{ℝ}}^{2}$ are image features, $X^{i}\in {\text{ℝ}}^{3}$ are world 3D points, for i∈S the set of all matches. ρ is the Cauchy cost function. π is the projection function. This minimization problem is solved iteratively using the Levenberg–Marquardt algorithm. Furthermore, outliers are detected and excluded and the optimization is run for a second time with the inlier set.

### 3.6. Local Map Maintenance

We maintain a secondary map of points we refer to as staged points, which are also tracked over time but are not used in motion estimation until they are found to be of high quality. After camera pose is computed, staged map points are updated. When a new 3D-points triangulation occurs, these new points are initially stored in this staging area and are not added immediately to the local map. If a staged point is tracked successfully for a specified number of frames, then it is declared of good quality and added to the map. If it fails tracking, then it is removed. However, if the number of map points drops below 1000 point, then staged points will be added immediately to the map in order to always maintain a minimum number of points in the map.

For the purpose of triggering a new points triangulation, we will monitor the number of 2D-3D matches found in the current frame and in the previous two frames. If the number of matches is decreasing, a new triangulation is performed. New points are triangulated from features that were not tracked in the current frame. These newly triangulated points are added to the staging area as described previously. Additionally, map points that fail tracking for a specified number of frames are deleted from the map. Hence, the local map is kept fresh with good immediately useful points from the staging area, while no-longer-trackable points are removed.

When the system initially starts, the local map is empty, so the first frame is used to triangulate the initial set of 3D points, which are added immediately to the local map. This first frame also sets the world reference coordinate frame. All reported poses will be with respect to this world coordinate frame. In the stereo case, triangulations are performed using the Linear-LS method described by Hartley and Sturm [25]. As the stereo frame is rectified, matching corners between the left and right images is greatly simplified, as the search is restricted to the same row in both images. In the RGB-D case, triangulations are performed by extracting depth values directly from the depth image.

### 3.7. General Implementation Remarks

The algorithm is implemented using the C++ programming language. OpenCV library was used to perform image processing tasks. Corner detector and descriptor extractor implementations available as part of OpenCV library were used. The general graph optimization (g2o) library [26] was used to perform the Levenberg–Marquardt minimization in the pose estimation step.

The algorithm runs purely on the CPU, with no GPU acceleration used. Moreover, the algorithm runs primarily in one thread. However, a parallel thread is spawned to perform the features computation step on the right image in the stereo cameras case while the main thread is busy performing it on the left one.

## 4. Evaluation

We present results of the evaluation of our visual odometry system on three challenging, publicly available datasets—namely, the KITTI dataset [27], the EuRoC MAV dataset [28] and the TUM RGB-D dataset [29]. Each dataset has its unique characteristics, which will enable comprehensive evaluation of our visual odometry system.

### 4.1. KITTI Dataset

The KITTI dataset is widely used for evaluating autonomous driving algorithms. The dataset was collected by driving in different traffic scenarios in the city of Karlsruhe, Germany. Some of the challenging aspects of the dataset are the presence of dynamic moving objects (vehicles, cyclists and pedestrians), the different lighting and shadow conditions as the vehicle is moving and the presence of foliage, which results in the detection of many non-stable and challenging-to-track corners.

The presented visual odometry system is also evaluated against two open-source systems, S-PTAM [11] and LIBVISO2 [30]. S-PTAM is a state-of-the-art full V-SLAM system. S-PTAM was compiled without loop-closing capability but all other operations remain intact. LIBVISO2 is a famous stereo visual odometry system that tracks features and estimates motion between consecutive frames only. With S-PTAM being a V-SLAM system and LIBVISO2 a frame-to-frame visual odometry system, they provide a good comparison ground for our presented system, which aims to reach V-SLAM systems accuracy while being lightweight like typical visual odometry systems.

#### 4.1.1. Accuracy

For evaluating the accuracy, two error metrics will be reported. First is the average translation error $E_{t}$ over all subsequences of length (100, …, 800) meters as defined in the KITTI dataset paper [27]. We define the other metric ξ as:

(8) $$\xi =\;RMSE\left(T_{1:n}\right)=\;{\left(\frac{1}{n}\overset{n}{\underset{i=1}{\sum }}T_{i}^{2}\right)}^{1/2}$$

where $T_{i}$ is the magnitude of the Euclidean distance along the horizontal plane between the estimated and ground truth pose at frame i.

**Figure 2:**
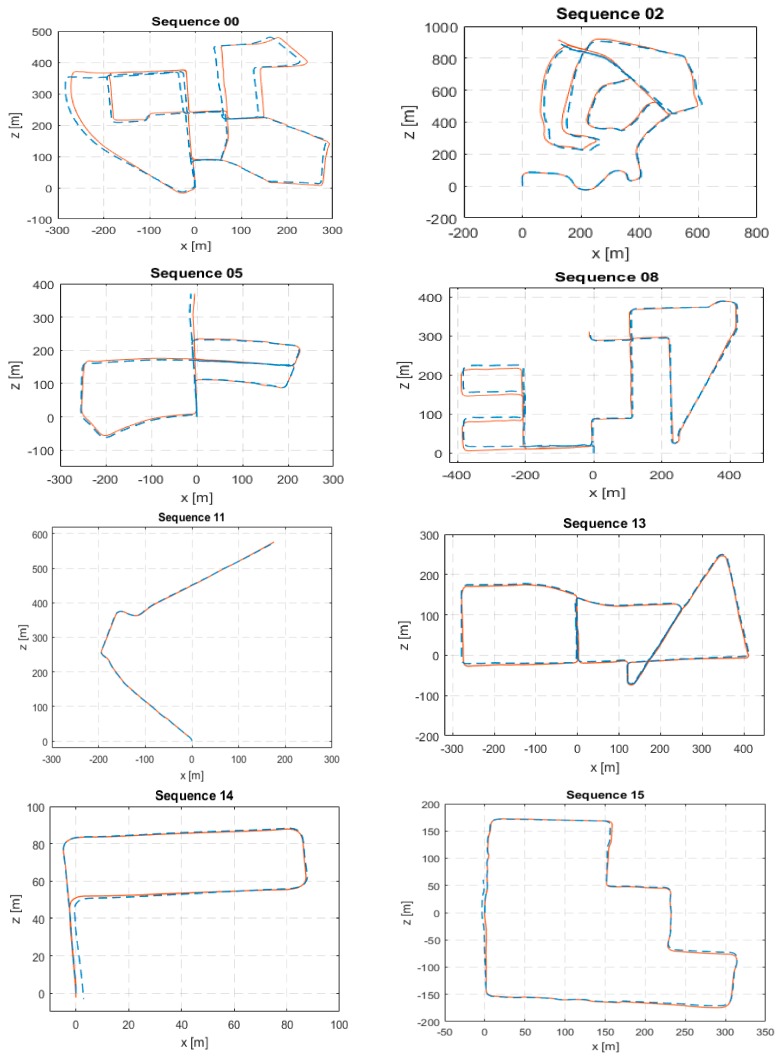
Estimated trajectory (dashed blue) from lightweight visual tracking (LVT) against ground truth (solid red) in KITTI dataset training sequences (00, 02, 05, 08) and evaluation sequences (11, 13, 14, 15).

The computed error metrics for KITTI sequences (00–10) except for sequence 01 are shown in Table 1. Sequence 01 is a challenging highway with unreliable far features. Although our algorithm does not lose tracking, it drifts badly and fails to provide meaningful estimates. Hence, it was excluded. From the presented results, it can be seen how our proposed visual odometry algorithm comes very close to S-PTAM, which is a complete V-SLAM system and surpasses LIBVISO2. Plots of the estimated path against ground truth are shown in Figure 2.

Additionally, we submitted our system for evaluation on the KITTI sequences (11–21) which are test sequences that have no publicly available ground truth. Our system achieved an average translation error of 5.8%. While running the analysis, we suspect the main source of error is sequence 21 which is a highway and suffers from the same issue as sequence 01 before. The problem with highway scenes is that features are far away relative to the stereo baseline causing the stereo cameras to degenerate into a monocular one. This results in the loss of scale information. Moreover, the KITTI evaluation server provides the path plots of a portion of the test sequences which are shown in Figure 2. From these path plots, we can see that our system is able to provide accurate estimates on the test sequences comparable to its estimates on the training ones. This means with the exclusion of the highway sequence 21, we expect our system to attain a comparable average translation error to the one achieved on the training sequences which is 1.23%. Properly handling the problematic far away features is a task for future work.

**Figure 3:**
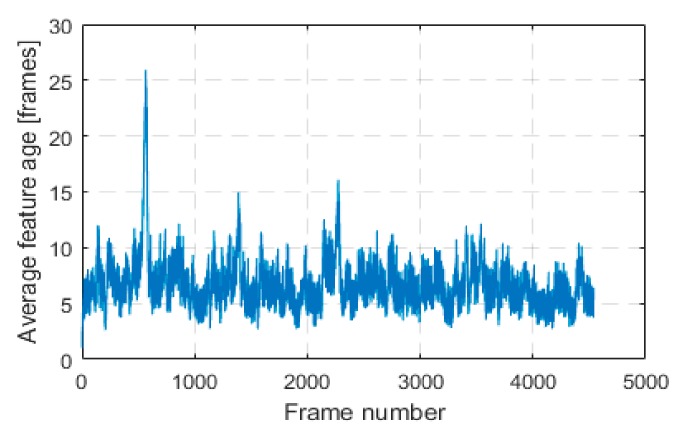
Average feature age in each frame of KITTI sequence 00.

An important observation is that through our innovative approach of keeping features alive in a local 3D map and tracking them for as long as possible, we were able to greatly improve estimation accuracy compared to LIBVISO2. LIBVISO2 follows the traditional visual odometry approach of tracking features between consecutive frames, that is, from frame to frame only. We will define feature age as the number of frames in which this feature was successfully tracked and used in pose estimation. Average feature age in each frame for KITTI sequence 00 is shown in Figure 3.

#### 4.1.2. Runtime Performance

Runtime performance evaluation experiments were performed on a laptop computer running the Ubuntu 16.04 operating system, with an Intel i7-7700HQ CPU and 16 GB of memory (RAM). For evaluating the runtime speed, we have timed the processing time of each frame from KITTI sequence 00, excluding the portion where the system is retrieving the stereo image and with no visualization enabled. As for evaluating the memory requirement, memory is read from the System Monitor utility just before the last frame. This process was repeated five times to account for operating system loading variability and the results are listed in Table 2.

**Figure 4:**
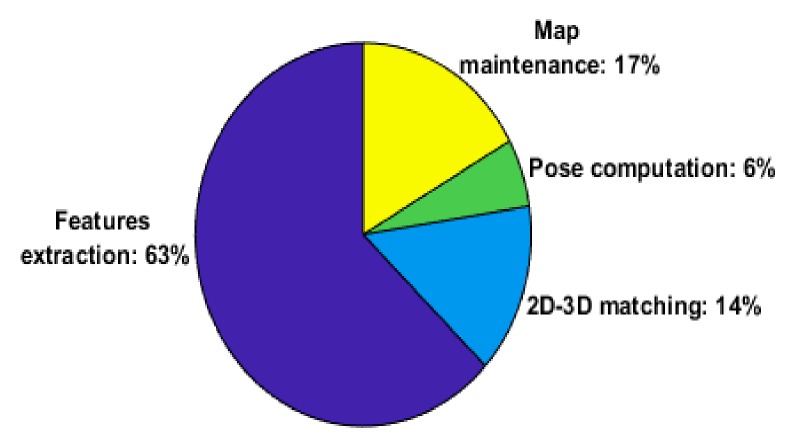
Relative runtime speed of the main stages of the visual odometry system.

We have timed the four main stages of the visual odometry system and the result is shown in Figure 4.

The suitability of the presented visual odometry system for constrained real-time applications is evaluated on two embedded Linux single-board computers, namely, the Raspberry PI 3 [31] and the ODROID XU4 [32]. The time to process each frame of KITTI sequence 00 is recorded and computational performance is listed in Table 3.

### 4.2. EuRoC Dataset

**Figure 5:**
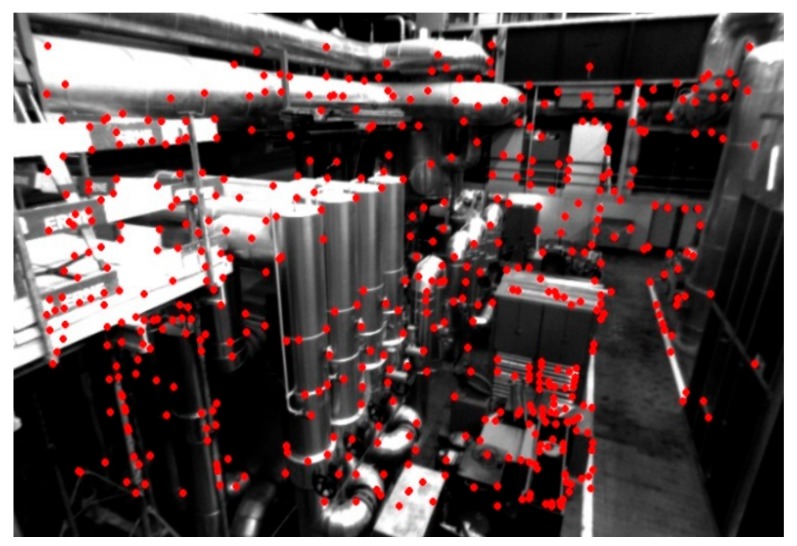
A sample image from Machine Hall 01 of the EuRoC datasets, where detected features are plotted (red dots).

The EuRoC datasets [28] were collected on board a micro-aerial vehicle (MAV). Stereo images were collected at a rate of 20 Hz with a stereo camera that provides monochrome WVGA images. The different datasets vary in their level of difficulty based on the flight dynamics and illumination conditions. The fact that AGAST corners and BRIEF descriptors used in LVT are by their design not invariant to in-plane rotations will provide useful insights, given that the MAV is moving in all 6 degrees of freedom. We will use the five sequences collected in an industrial machine hall for evaluation. The VICON room sequences constitute primarily of white plain surfaces and there is not enough texture in the scene for the feature detector to detect corners, thus they were not used in the evaluation. A sample image from the first machine hall dataset showing the detected features is shown in Figure 5.

#### 4.2.1. Accuracy

For evaluating the accuracy, two error metrics, absolute trajectory error (ATE) and relative pose error (RPE), as defined Sturm et al. [29], are used. The ATE is well suited for evaluating Visual SLAM systems, as it measures the global consistency of the estimated trajectory. On the other hand, the RPE is more suited for evaluating visual odometry systems, as it measures the local accuracy of a trajectory over a fixed time interval. That is, it measures the drift in a trajectory. In our evaluation, as the stereo images are recorded at a rate of 20 Hz, we will set the fixed time interval for RPE to be 0.05 s. Therefore, RPE will correspond to drift in units of meters per second. We have also run LIBVISO2 [30] on the same dataset. The results are reported in Table 4. Both visual odometry systems are achieving similar drift rates.

#### 4.2.2. Effect of Rotational-Invariant Features

AGAST corners and BRIEF descriptors used in our presented visual odometry system do not provide rotational invariance capability. When evaluated previously on the KITTI dataset, this did not pose a problem, as the vehicle is moving on a locally planar ground. However, in the EuRoC datasets, the MAV is flying in all 6 degrees of freedom. In order to evaluate said effect, we have replaced the default AGAST/BRIEF with oriented fast and rotated brief (ORB) [33]. ORB provides rotational-invariant features. With everything else remaining fixed, we have re-run the evaluation and the results are reported in Table 5.

From Table 5, with the same original parameters, LVT now fails to complete the MH_02_easy dataset. The cause is that the system fails to track enough ORB features in a motion-blurred frame. Drift rates are almost the same and no clear improvement in accuracy is attained by the new feature detector alone. We found from this experiment that simply replacing the feature detector with a rotationally-invariant one did not result in any major improvement in the results as was expected.

### 4.3. TUM RGB-D Dataset

The Technical University of Munich (TUM) RGB-D dataset [29] is a large dataset collected indoors using an RGB-D sensor under different illumination, texture and movement scenarios. The data was collected at a 30 Hz frame rate and a sensor resolution of 640 × 480. The evaluation results on a subset that is suited for V-SLAM and visual odometry evaluation are reported in Table 6 along with an evaluation against Fovis. Although our visual odometry system was initially designed for stereo cameras, it was, surprisingly, able to achieve low drift rates on RGB-D data. The main difference is that we found it necessary to trigger a new triangulation operation at every frame, unlike the default behavior as described in map maintenance before. We can see from Table 6 that our system is able to achieve similar drift rates to Fovis which was originally designed for RGB-D cameras. However, a fast rotation while the camera faces a low-textured wall during the movement results in the error in fr1_room for our system.

## 5. Conclusions and Future Work

In this paper, we have presented our feature-based visual odometry system called LVT, which is compatible with both stereo and RGB-D sensors. Its innovative usage of a transient local map enables it to approach the estimation accuracies common to full V-SLAM systems. The algorithm is designed for real-time operation with low computational overhead and memory requirements. The system was evaluated on KITTI autonomous driving datasets and compared with state-of-the-art V-SLAM and VO systems. Furthermore, it was also evaluated on the EuRoC MAV industrial machine hall and TUM RGB-D datasets as other use cases. This paper accompanies the release of the source code of the system under a permissive license and with support for the Robot Operating System (ROS). The source package contains the example codes used to run our system on the three datasets used in the evaluation along with the used parameters.

Visual SLAM and odometry approaches can be classified as either monocular when one camera is used or stereo when more than one is used. One of the major issues in monocular methods is the scale ambiguity, which means that motion trajectory can be estimated with only an ambiguous scale factor. On the other hand, with a known baseline distance between cameras, stereo methods can estimate the exact motion trajectory, that is, they are able to recover global metric scale. Another issue with monocular methods is that they require careful initialization procedures and usually involve tricky ones, whereas in stereo methods it is easier to achieve a power-and-go system. The main downside of stereo methods is that they degenerate into the monocular case for scenes with very distant objects relative to the stereo camera baseline. This downside was the main failure cause for the KITTI sequence 01, which consists of a highway with far features dominating. Properly handling far away features should be addressed in future work. The loss of scale for highway scenes in our algorithm occurs because 3D point placement is based entirely on stereo disparity. Increasing the stereo camera baseline and/or resolution could provide better depth measurement and address this challenge. Moreover, as far points have large uncertainty in their placement, re-initializing them in subsequent frames is expected to contribute to this problem solution. Another solution would be to use other sensor modalities to estimate scale, such as integrating inertial sensors, or using other odometry techniques based on wheel odometers or LIDAR.

In our experience, building a visual odometry system is tricky as there are many heuristics involved in each part of it. We aimed to build a framework that is adaptable and extensible. For example, switching different feature extractors can be done easily in our system. Furthermore, as the system was initially designed for stereo cameras, the ease of extending it to RGB-D cameras was a pleasant surprise. We expect that extending the system to additional specialized depth sensors in future work, such as infrared (IR) cameras, to be easy as well.

Some challenging frames, such as abrupt movements, lighting changes and low texture, might result in poor pose estimates by visual odometry or even complete loss of tracking. A mechanism to bridge these moments of disruptions is a potential future work. Integrating with an IMU could provide such a mechanism. Additionally, it could reduce drift accumulated by visual odometry.

## Figures and Tables

**Table 1:** Results on KITTI dataset.

Sequence	Length (km)	LVT		S-PTAM		LIBVISO2	
		*E_t_* (%)	ξ (m)	*E_t_* (%)	ξ (m)	*E_t_* (%)	ξ (m)
00	3.7223	1.25	11.01	0.84	8.81	2.74	47.03
02	5.0605	1.33	13.59	0.96	22.20	2.20	69.52
03	0.5590	1.04	2.48	1.14	3.43	2.27	4.66
04	0.3936	0.56	0.78	1.29	2.72	1.08	2.67
05	2.2046	0.89	4.27	0.91	3.01	2.26	19.80
06	1.2326	1.04	2.11	1.28	3.34	1.28	4.20
07	0.6944	0.98	3.17	0.88	2.97	2.34	5.74
08	3.2137	1.25	6.57	1.05	6.69	2.83	44.52
09	1.7025	1.76	9.23	1.21	9.28	2.84	24.32
10	0.9178	1.02	3.81	0.64	3.61	1.39	2.97
Total	19.701	1.23	9.05	0.97	11.73	2.45	43.98

**Table 2:** Runtime performance measurements.

Algorithm	Mean ± Std (ms)	Memory
LVT	13.82 ± 4.4	22.12 MiB
S-PTAM	36.7 ± 23.8	1.5 GiB
LIBVISO2	23.2 ± 4.5	41.82 MiB

**Table 3:** Computational performance on embedded computers.

Single-Board Computer	Mean ± Std (ms)
Raspberry Pi 3	162.37 ± 42.38
ODROID XU4	87.96 ± 20.35

**Table 4:** RMSE values of absolute trajectory error (ATE) and relative pose error (RPE) on EuRoC datasets.

	LVT		LIBVISO2	
Sequence	ATE (m)	RPE (m/s)	ATE (m)	RPE (m/s)
MH_01_easy	0.232	0.028	0.234	0.028
MH_02_easy	0.129	0.028	0.284	0.028
MH_03_medium	1.347	0.070	0.86	0.069
MH_04_difficult	1.635	0.069	1.151	0.068
MH_05_difficult	1.768	0.060	0.818	0.060

**Table 5:** RMSE values of ATE and RPE on EuRoC datasets using rotational-invariant oriented fast and rotated brief (ORB) features.

Sequence	ATE (m)	RPE (m/s)
MH_01_easy	0.292	0.029
MH_02_easy	x	x
MH_03_medium	1.328	0.071
MH_04_difficult	2.194	0.070
MH_05_difficult	1.515	0.061

**Table 6:** RMSE values of ATE and RPE on TUM RGB-D dataset.

	LVT		Fovis	
Sequence	ATE (m)	RPE (m/s)	ATE (m)	RPE (m/s)
fr1_desk	0.109	0.010	0.259	0.009
fr1_desk2	0.116	0.012	0.125	0.009
fr1_room	4.138	0.077	0.184	0.007
fr1_xyz	0.035	0.006	0.051	0.006
fr2_desk	0.080	0.003	0.103	0.003
fr2_xyz	0.014	0.002	0.013	0.002
fr3_office	0.132	0.006	0.188	0.005
